# Inflammaging and the sex-frailty paradox

**DOI:** 10.1007/s40520-025-03181-7

**Published:** 2025-10-07

**Authors:** Beatrice Arosio, Greta Salafia, Evelyn Ferri, Daniela Mari, Eleonora Tobaldini, Giovanni Vitale, Nicola Montano

**Affiliations:** 1 Department of Clinical Sciences and Community Health, Dipartimento di Eccellenza 2023–2027, University of Milan, Via della Commenda 19, Milan, 20122 Italy; 2 Fondazione IRCCS Ca’ Granda Ospedale Maggiore Policlinico, Via Francesco Sforza 35, Milan, 20122 Italy; 3 Laboratory of Geriatric and Oncologic Neuroendocrinology Research, IRCCS, Istituto Auxologico Italiano, Via Zucchi 18, Cusano Milanino, 20095 Italy; 4 Department of Medical Biotechnology and Translational Medicine, University of Milan, Via Vanvitelli 32, Milan, 20133 Italy

**Keywords:** Aging, Frailty, Gender-differences, Inflammation, Cytokines

## Abstract

**Background:**

The “sex-frailty paradox” is a concept that indicates how women despite being more frail are less susceptible to death than men. The roots of this paradox may lie in the different combination of biological, behavioural, and social factors between the two sexes. The aim of this study is to deepen our understanding of the different biological mechanisms underlying frailty and longevity in men and women, thus shedding further light on the sex-frailty paradox.

**Methods:**

We studied 452 subjects (315 women and 137 men) stratifying them by age (≤ 80, 81–99 and ≥ 100 years) and sex. A 47-item frailty index was calculated. Plasma concentrations of inflammatory markers were analysed by next-generation ELISA.

**Results:**

Women aged ≤ 80 years were less frail while those aged ≥ 100 years were more frail than their male counterparts. Interestingly, the 81-99-year-old group showed similar frailty degree between females and males. Most of the biomarkers increased with age in both sexes, while being associated differently with frailty, indicating that there are specific biological roots in the two sexes that influence frailty.

**Conclusions:**

Trying to delineate the unique molecular profile of older women and men becomes essential for understanding the mechanisms underlying the sex-frailty paradox. Further research on sex-specific determinants is required to enhance our understanding of aging and develop effective strategies to promote health and longevity in both sexes.

**Supplementary Information:**

The online version contains supplementary material available at 10.1007/s40520-025-03181-7.

## Introduction

The United Nations Department of Economics and Social Affairs estimates that by 2050 approximately 21.3% of the world’s population will be 60 years of age or older [1]. Interestingly, a notable disparity in life expectancy between women and men exists, with older women outnumbering older men [2].

Aging is an unavoidable process involving a series of complex changes that take place within the human body and require continuous responses and adaptations to stimuli throughout life [3]. This process stems from the accumulation of molecular and cellular damage, influenced by social, behavioral, psychological, environmental, epigenetic and genetic mechanisms [4], which affect the functionality of various systems and organs [5].

In particular, during aging, the immune cell functionality gradually decreases, resulting in a phenomenon known as “immunosenescence”, which undermines both the innate and adaptive immune systems, leading to an increased incidence of disease and infection [6, 7].

Immunosenescence is the basis of inflammaging, a phenomenon that occurs during aging and consists of a chronic and persistent state of low-grade inflammation characterized mainly by the production of components of the innate immune response [8]. The theory posits that excessive stimulation of pro-inflammatory pathways and an ineffective anti-inflammatory response are a driving force behind the development of frailty and age-related diseases [9–11].

Specifically, frailty reflects a state of increased vulnerability to stressors, a consequence of the gradual decline in the individual’s homeostasis and functional reserves [12]. A sex-associated divergence in frailty and mortality, termed the “sex-frailty paradox”, is widely known [13]. This consists of the observation that women, while generally living longer than men, often show higher rates of frailty reflecting a worse health status [14, 15]. This could be due to the fact that men tend to suffer from more malignant conditions (e.g. stroke and ischaemic heart disease), while women mainly from “life-threatening” chronic conditions associated with greater morbidity (e.g. fractures, constipation, depression and headaches) [16].

Research on sex-specific differences in frailty and its contributing factors suggests that these disparities are probably the result of the complex interplay between biological, psychosocial and behavioural factors which differ between women and men [17]. Interestingly, some studies have shown a different association between certain inflammatory markers and frailty in women and men [18, 19]. In this context and with the aim of characterising the aging continuum, we have recently shown that plasma concentrations of inflammatory biomolecules linked to inflammation and neuroinflammation, such as interleukin (IL)-6, tumour necrosis factor (TNF)-α, TNF receptor 1 (TNFR1), and soluble triggering receptors expressed on myeloid cells (sTREM-1 and sTREM-2) were differentially associated with age, frailty and sex in a cohort of older and very old individuals [20].

In this study, using the same cohort of patients [20], we examined different age groups, investigating the influence of the sex-frailty paradox on the biological foundations of aging. Our aim was to better understand the different biological mechanisms underlying frailty and longevity in men and women, thereby shedding further light on the sex-frailty paradox.

## Materials and methods

### Study design

Subjects were recruited as part of a cohort study conducted in Northern Italy between 2012 and 2022. Characteristics of this cohort have already been published [20]. A total of 452 subjects (315 females and 137 males aged 43 to 114 years) with all the necessary information for calculating the frailty index (FI) and with frozen biological samples available for biomarker analysis were included in the study.

Briefly, when participants were enrolled in the study, a team of specialists administered a questionnaire to capture data on their health, functional (Activities of Daily Living, ADL) and cognitive status (Mini-Mental State Examination, MMSE), depression (Geriatric Depression Scale, GDS), drugs assumption, medical history and lifestyle habits (Body Mass Index, BMI) [21]. Moreover, the presence of certain signs and symptoms (e.g. pain, bowel incontinence, sleep disturbances, edema, and tremor) was also recorded.

Participants with diagnosed acute or chronic inflammatory diseases, such as rheumatoid arthritis, lupus, psoriasis, asthma, autoimmune diseases, Crohn’s disease, multiple sclerosis and ulcerative colitis, and with Alzheimer’s disease (AD) [22] and vascular dementia [23] were systematically excluded from the study. Finally, patients who were receiving anti-inflammatory therapy for several days at the time of recruitment were also excluded.

The research protocol was approved by the Ethical Committee of the Fondazione IRCCS Ca’ Granda Ospedale Maggiore Policlinico, Milan (Prot. n. 2035, amendment 30/11/2011). Only individuals who gave written informed consent were included in the study.

### Frailty index

A FI was assessed as reported in our previous study [20]. Briefly, each variable received a score of 0 indicating no deficit and a score of 1 indicating the presence of a deficit. The FI was obtained by dividing the number of health deficits of the individual by the 47 variables considered for its calculation (Supplementary Table 1) [20, 24, 25]. Participants with more than 30% missing variables were omitted.

### Plasma samples analysis

At recruitment, fasting blood samples were taken in Ethyl-enediaminetetraacetic acid (EDTA) tubes and centrifuged at 1200 g for 15 min at room temperature. The platelet-free plasma was frozen and stored at -80 °C until quantification of the analytes.

We measured the plasmatic concentrations of interferon (IFN)-γ, IL-10, IL-6, IL-1β, TNF-α, TNFR1, sTREM-1 and sTREM-2, and neurofilament light chain (NfL) by Human Simple Plex assays (ProteinSimple, CA, USA) on Ella instrument (Bio-Techne, Minneapolis, MN, USA).

### Statistical analysis

Statistical analyses were performed with IBM SPSS Statistics software (version 28, IBM Inc., Chicago, IL, USA). The Kolmogorov-Smirnov test was used to assess the distribution of the variables. Age, being normally distributed, was expressed as mean and standard deviation (SD), whereas variables such as FI and marker concentrations, which did not follow a normal distribution, were presented as median and interquartile range (IQR: 25–75th percentile). Sex was reported as percentage.

Comparisons were performed using the Mann-Whitney’s U-test and the Kruskal-Wallis test. Multiple linear regression analysis was conducted to explore the associations between log-transformed concentrations of all analyzed markers (dependent variable) and age and log-transformed FI scores (independent variables). A *p*-value of less than 0.05 was used to determine statistical significance.

## Results

As previously described, the mean age of the entire cohort was 79.7 years (SD 11.2, 43 to 114 years), women accounted for 69.8% and the median FI value was 0.19 (IQR 0.12–0.29, 0.00 to 0.75) [20].

When we stratified the enrolled population into three age groups, the median FI was 0.16 (IQR 0.09–0.24) in people aged less than or equal to 80 years (aged ≤ 80 years, 194 women and 82 men), 0.23 (IQR 0.16–0.30) in people aged between 81 and 99 years (aged 81–99 years, 82 women and 45 men), and 0.54 (IQR 0.48–0.58) in people aged 100 years or older (aged ≥ 100 years, 39 women and 10 men), remarking that the FI increased with age (*p* < 0.001).

Considering women and men separately, in people aged ≤ 80 years the FI value was significantly lower in women than in men. Conversely, in people aged ≥ 100 years the FI value was higher in women, with a difference that was nearly significant (*p* = 0.06). Interestingly, no difference was found between the two sexes in people aged 81–99 years (Table 1).

**Table 1:** FI values in women and men aged ≤ 80 years, 81–99 years and ≥ 100 years

	Groups	Women	Men	*p*
FI	≤ 80 years	0.14 (0.09–0.21)	0.21 (0.14–0.28)	**< 0.001**
	81–99 years	0.22 (0.14–0.29)	0.24 (0.18–0.31)	0.14
	≥ 100 years	0.55 (0.48–0.61)	0.48 (0.41–0.54)	0.06

FI values were expressed as median (IQR). IQR, interquartile range; FI, Frailty index

Men aged ≤ 80 years showed significantly higher concentrations of IL-10, IL-6, TNF-α and sTREM-1 than women of the same age. The concentrations of IL-6, TNFR1 and sTREM-1 were also higher in men than in women aged 81–99 years (Table 2). In centenarians, NfL concentrations were significantly decreased in men (Table 2).

**Table 2:** Plasma concentrations of markers in women and men aged ≤ 80 years, 81–99 years and ≥ 100 years

Biomarkers	Groups	Women (*n* 315)	Men (*n* 136)	*p*
IFN-γ (pg/mL)	≤ 80 years	0.71 (0.49–1.06)	0.65 (0.43–1.01)	0.26
	81–99 years	0.67 (0.49–1.14)	0.67 (0.49–0.96)	0.86
	≥ 100 years	1.34 (0.71–2.11)	0.88 (0.68–1.46)	0.28
IL-10 (pg/mL)	≤ 80 years	1.83 (1.44–2.34)	2.07 (1.55–2.64)	**0.04**
	81–99 years	2.25 (1.73–3.05)	2.36 (1.70–3.68)	0.35
	≥ 100 years	3.69 (2.99–4.43)	3.94 (1.88–7.23)	0.80
IL-6 (pg/mL)	≤ 80 years	2.23 (1.53–3.22)	2.61 (1.68–4.39)	**0.04**
	81–99 years	3.21 (2.05–5.33)	4.26 (2.68–6.19)	**0.04**
	≥ 100 years	12.3 (7.54–21.8)	19.3 (5.69–54.4)	0.57
IL-1β (pg/mL)	≤ 80 years	0.17 (0.09–0.27)	0.21 (0.12–0.31)	0.15
	81–99 years	0.20 (0.10–0.31)	0.22 (0.12–0.36)	0.50
	≥ 100 years	0.48 (0.23–0.86)	0.68 (0.39–0.94)	0.32
TNF-α (pg/mL)	≤ 80 years	8.61 (7.34–10.3)	9.36 (7.60–11.6)	**0.02**
	81–99 years	10.1 (8.26–12.7)	10.9 (9.89-13.0)	0.11
	≥ 100 years	18.3 (14.3–21.7)	19.4 (18.1–26.5)	0.14
TNFR1 (ng/mL)	≤ 80 years	1.26 (1.06–1.47)	1.31 (1.09–1.64)	0.07
	81–99 years	1.55 (1.29–1.99)	1.82 (1.61–2.29)	**0.004**
	≥ 100 years	3.31 (2.24-4.00)	3.44 (2.48–4.49)	0.55
sTREM-1 (ng/mL)	≤ 80 years	0.45 (0.37–0.55)	0.48 (0.40–0.63)	**0.02**
	81–99 years	0.61 (0.42–0.76)	0.67 (0.53–0.88)	**0.03**
	≥ 100 years	1.00 (0.71–1.39)	1.11 (0.89–1.52)	0.26
sTREM-2 (ng/mL)	≤ 80 years	33.5 (25.1–44.2)	28.0 (21.0-40.5)	0.06
	81–99 years	42.4 (32.4–55.0)	42.6 (30.8–55.4)	0.80
	≥ 100 years	56.6 (44.4–75.2)	75.4 (39.9–100)	0.63
NfL (pg/mL)	≤ 80 years	28.6 (22.8–37.9)	27.1 (20.5–37.4)	0.31
	81–99 years	41.9 (32.6–57.6)	47.4 (42.0-58.1)	0.10
	≥ 100 years	118 (101–181)	93.4 (63.2–123)	**0.04**

Plasma concentrations of markers were expressed as median (IQR). IQR, interquartile range; IFN-γ, Interferon- γ; IL-10, Interleukin-10; IL-6, Interleukin-6; IL-1β, Interleukin-1β; TNF-α, Tumour necrosis factor-α; TNFR1, Tumour necrosis factor receptor 1; sTREM, Soluble triggering receptor expressed on myeloid cells; NfL, Neurofilament light chain

In women, multiple linear regression analysis revealed that age was positively associated with plasma concentrations of all markers analyzed, except for IFN-γ and IL-1β (Table 3). A strong positive association was also found between FI and IFN-γ, TNF-α and sTREM-2 concentrations, whereas this association was still significant but weaker for IL-6 and TNFR1 (Table 3).

In men, age was positively associated with the plasma concentrations of all markers investigated, with the exception of IFN-γ and IL-10 (Table 4). Additionally, higher concentrations of IL-6, TNFR1, and NfL were associated with higher FI values (Table 4).

**Table 3:** Multiple linear regression analysis between markers’ concentrations (dependent variable) and age and frailty index (FI) (independent variables) in women

	Covariates	*R* ^2^	B	SE	*p*
IFN-γ (pg/mL)	age	0.06	-0.003	0.005	0.56
	FI		0.28	0.08	**< 0.001**
IL-10 (pg/mL)	age	0.13	0.01	0.003	**< 0.001**
	FI		0.07	0.05	0.17
IL-6 (pg/mL)	age	0.26	0.03	0.005	**< 0.001**
	FI		0.16	0.08	**0.04**
IL-1β (pg/mL)	age	0.01	0.002	0.008	0.81
	FI		0.14	0.11	0.21
TNF-α (pg/mL)	age	0.34	0.01	0.002	**< 0.001**
	FI		0.12	0.03	**< 0.001**
TNFR1 (ng/mL)	age	0.37	0.02	0.002	**< 0.001**
	FI		0.07	0.03	**0.03**
sTREM-1 (ng/mL)	age	0.19	0.02	0.003	**< 0.001**
	FI		0.02	0.05	0.61
sTREM-2 (pg/mL)	age	0.27	0.01	0.003	**< 0.001**
	FI		0.14	0.04	**< 0.001**
NfL (pg/mL)	age	0.48	0.04	0.003	**< 0.001**
	FI		0.04	0.05	0.41

Markers’ concentrations and frailty index were log-transformed. R^2^, Coefficient of determination; B, Unstandardized β coefficient; SE, Standard error for the unstandardized β coefficient; FI, Frailty index; IFN-γ, Interferon- γ; IL-10, Interleukin-10; IL-6, Interleukin-6; IL-1β, Interleukin-1β; TNF-α, Tumour necrosis factor-α; TNFR1, Tumour necrosis factor receptor 1; sTREM, Soluble triggering receptor expressed on myeloid cells; NfL, Neurofilament light chain

**Table 4:** Multiple linear regression analysis between markers’ concentrations (dependent variable) and age and frailty index (FI) (independent variables) in men

	Covariates	*R* ^2^	B	SE	*p*
IFN-γ (pg/mL)	age	0.05	0.003	0.008	0.67
	FI		0.25	0.13	0.06
IL-10 (pg/mL)	age	0.10	0.01	0.007	0.10
	FI		0.20	0.11	0.06
IL-6 (pg/mL)	age	0.33	0.04	0.008	**< 0.001**
	FI		0.37	0.14	**0.008**
IL-1β (pg/mL)	age	0.08	0.04	0.01	**0.002**
	FI		-0.20	0.20	0.33
TNF-α (pg/mL)	age	0.37	0.02	0.003	**< 0.001**
	FI		0.09	0.05	0.08
TNFR1 (ng/mL)	age	0.52	0.02	0.003	**< 0.001**
	FI		0.19	0.05	**< 0.001**
sTREM-1 (ng/mL)	age	0.33	0.02	0.004	**< 0.001**
	FI		0.09	0.06	0.13
sTREM-2 (pg/mL)	age	0.28	0.03	0.005	**< 0.001**
	FI		0.09	0.08	0.25
NfL (pg/mL)	age	0.45	0.04	0.005	**< 0.001**
	FI		0.23	0.09	**0.01**

Markers’ concentrations and frailty index were log-transformed. R^2^, Coefficient of determination; B, Unstandardized β coefficient; SE, Standard error for the unstandardized β coefficient; FI, Frailty index; IFN-γ, Interferon- γ; IL-10, Interleukin-10; IL-6, Interleukin-6; IL-1β, Interleukin-1β; TNF-α, Tumour necrosis factor-α; TNFR1, Tumour necrosis factor receptor 1; sTREM, Soluble triggering receptor expressed on myeloid cells; NfL, Neurofilament light chain

## Discussion

The key finding of this study is the sex-specific association observed between markers of inflammation, neuroinflammation, and neurodegeneration and frailty, throughout aging, pointing to gender-specific biological roots in the sex-frailty paradox [26]. Interestingly, women and men in this cohort showed different levels of frailty across aging. Women aged ≤ 80 years were less frail compared to their male peers, while women and men aged 81–99 years showed a similar degree of frailty. Finally, according to data on centenarians [26], centenarian women were more frail than their male counterparts.

In our cohort, men aged ≤ 80 years showed significantly higher FI values than women of the same age, suggesting that their aging trajectory up to their average life expectancy (81.1 years, 27) reflects a greater susceptibility to developing chronic diseases (e.g. heart disease, arteriosclerosis and emphysema) earlier than women [28, 29]. Our result was in disagreement with several frailty studies conducted on community-dwelling older populations, which reported higher FI values in women than in men [30–32]. The male-female health-survival paradox has yielded inconsistent results across studies examining frailty [32–36]. Indeed, despite the fact that the FI is able to capture this paradox well, the average FI values (by age group) vary significantly [37]. These discrepancies may stem from inherent differences in sample populations, including gender roles, ethnicity, socio-cultural factors, and healthcare systems, which are likely to contribute to the heterogeneity observed across studies.

Interestingly, no significant difference in frailty was found between women and men in the 81–99 age group. This narrowing of the gap in FI scores between the two sexes may be consistent with the sex-frailty paradox. In fact, while men acquire fewer deficits beyond the age of 80 and the most frail tend to die earlier, as women age they rapidly develop chronic diseases and disabilities, but survive thanks to their great resilience [37].

As expected, centenarian women exhibited higher FI values compared to men, further corroborating the sex-frailty paradox; however, this difference did not reach statistical significance (*p* = 0.06), probably due to the limited sample size of men in this group (26%).

The observed differences in FI values between women and men in the three age groups paralleled the peculiar associations of biomarker concentrations. This finding was in agreement with the concentrations of IL-10 and TNF-α, which were higher in men aged ≤ 80 years, and with the concentrations of IL-6 and sTREM1, which were higher in men aged ≤ 80 as well as in men aged 81–99 years than in women peers. TNFR1 concentrations were also higher in both men aged ≤ 80 and men aged 81–99 than women of the same age, although in the youngest group they only showed a trend close to significance. These data may support the fact that immune-senescence is accelerated in men compared to women [38, 39], resulting in a greater decrease in B lymphocytes and naïve T lymphocytes and a greater increase of memory T lymphocytes and natural killer cells [38]. This alteration of immune cell function results in elevated levels of pro-inflammatory as well as anti-inflammatory cytokines, the so-called inflammaging, and in immune system dysfunction, which may underlie reduced longevity in men [38].

Although the concentrations were generally higher in men, the associations between IL-6, TNF-α, and TNFR1 levels with age and FI were observed in both sexes. However, in men, TNF-α concentrations showed only a trend in the association with FI. The strongest association between TNF-α and FI in women could be due to the fact that this cytokine is associated with pathological conditions [40–44] that seem to be more prevalent in women [17]. In contrast, the strong association observed in men between IL-6 and TNFR1 concentrations and FI could be related to the more rapid inflammaging seen in men, even within our cohort up to 99 years of age, which potentially increases the risk of developing more severe complications [45, 46] and fatal age-related diseases [47]. These results aligned with the trend approaching significance observed in men regarding the association between IL-10 and FI, confirming that the anti-inflammatory response was closely linked with the pro-inflammatory one. In contrast, in women, a strong association was observed between IL-10 concentrations and age, in agreement with already published data showing that the production of this cytokine gradually increases with decreasing estrogen levels [48].

In the group of centenarians, however, we observed no differences in markers related to the inflammatory response, whereas we observed significantly lower plasma concentrations of NfL in men than in women. These findings might reflect the ability of long-lived individuals to activate appropriate anti-inflammatory pathways aimed at optimizing the balance between pro- and anti-inflammatory cytokines and thus contributing to their longevity [49, 50], regardless of the frailty status and sex of the individuals.

With reagard to NfL, concentrations of this marker were strongly associated with age in both sexes, as already described in the literature [51], probably reflecting the progressive axonal degeneration associated with normal aging [52]. Interestingly, NfL concentrations were only found to be positively associated with FI in men, demonstrating that several chronic diseases more prevalent in men, such as peripheral artery disease, heart failure, atrial fibrillation, and incident stroke [53–56] may influence its release. Indeed, NfL concentrations were significantly lower in centenarian men than in centenarian women, probably due to their better health status.

Finally, no significant differences were found between women and men in the three age groups in the concentrations of IFN-γ, IL-1β and sTREM2. IFN-γ may have a key role in frailty [20], as indicated by regression analyses that showed a strong association between IFN-γ concentrations and FI in women and a trend in men. This is consistent with a study that found increased plasma concentrations of this cytokine in frail, septuagenarian Italian women [57]. Although no difference was observed in the three age groups, IL-1β concentrations were found to be significantly associated with age in men, probably as a consequence of the gradual decline in androgen levels during aging, which is linked to increased IL-1β concentrations [58, 59]. In contrast, sTREM2 concentrations were positively associated with age in both sexes and with FI only in women, in whom a potential regulatory role of estrogen on this receptor was hypothesized [60].

Finally, sTREM-1 concentrations were also positively associated with age in both sexes, reinforcing the idea that the soluble form of these receptors increases with age [20]. Furthermore, the higher concentrations observed in men compared to women in both age groups ≤ 80 and 81–99 years could reflect a more pronounced inflammaging experienced by men.

One of the limitations of this study is the relatively small number of men that, despite being in line with epidemiological data on the world population, could compromise the representativeness of the results. Furthermore, to investigate inflammaging, we had to exclude all individuals suffering from major chronic inflammatory diseases, thus losing a substantial fraction of the older population and introducing a selection bias, that limits the applicability of the results in the clinical practice. Finally, the cross-sectional design of the study limits the ability to draw definitive conclusions regarding causality, as the observed results cannot be attributed exclusively to the health determinants examined, nor do they take into account the possible impact of unmeasured lifestyle factors. Unfortunately, since we did not have data on lifestyle habits (such as smoking or nutrition) for all subjects in the cohort, we were unable to adjust the results for these factors as well.

In conclusion, despite the abundance of existing studies, knowledge about the biology of sex differences in older people, taking into account frailty, is still scarce. Therefore, trying to delineate the molecular profile that characterizes older women and men becomes essential to better understand the underlying mechanisms that might explain the phenomenon of the sex-frailty paradox. Further research focusing on sex-specific determinants is needed to support the development of more effective strategies to promote health and longevity in both sexes and to tailor interventions to the specific needs of women and men.

## Supplementary Information

Below is the link to the electronic supplementary material.

Supplementary Material 1

## Data Availability

The data supporting the findings of this study are available from the corresponding author upon reasonable request.
