# Supplementary material for: Inflammaging and the sex-frailty paradox
Source: Aging Clin Exp Res. 2025 Oct 7;37(1):282. doi: 10.1007/s40520-025-03181-7 (PMC12504336; doi:10.1007/s40520-025-03181-7)
Supplement: Supplementary file 1 — Supplementary Material 1 [file 40520_2025_3181_MOESM1_ESM.docx]

**Supplementary Table 1.** List of the 47 biochemical and health deficits included in the FI.

| **Deficit** |
| --- |
| **Biochemical parameters**  Cholesterol >200 mg/dL  CRP >0.5 mg/dL  Vitamin B12 <191 ng/L and >663 ng/L  Folate <4.6 µg/L and >18.7 µg/L  25-OH Vitamin D <30 µg/L  TSH <0.28 mIU/L and >4.30 mIU/L  **Signs**  Pain  Bowel incontinence  Sleep disorders  BMI <21 kg/m^2^ and >30 kg/m^2^  Edema  Tremor  **Disabilities**  Mobility impairment  ADL - disability in self-feeding  ADL - disability in dressing  ADL - disability in bathing  ADL - disability in transferring  ADL - disability in toileting  ADL - incontinence  IADL – disability in using telephone  IADL - disability in shopping  IADL - disability in food preparation  IADL - disability in housekeeping  IADL - disability in doing laundry  IADL - disability in travelling by car or public transportation  IADL - disability in medication use  IADL - disability in handling finances  **Diseases**  Hypertension  Diabetes  Congestive heart failure  Coronary heart disease  Cardiac arrhythmia  Chronic obstructive pulmonary disease  Decreased visual acuity  Hearing loss  Osteoarthritis  Vascular endothelial abnormalities  Chronic renal insufficiency  Hepatopathy  Depression  Cerebrovascular disease  Cognitive impairment  Cancer  Osteoporosis  Anemia  Diverticulosis  Mild cognitive impairment |

CRP: C - reactive protein, TSH: Thyroid-Stimulating Hormone, BMI: Body Mass Index, ADL: Activity of Daily Living, IADL: Instrumental Activity of Daily Living.
